# 

**Published:** 1890-06-14

**Authors:** 


					I, 'lllfgll " r" V7:-: --
o, _r**~ ..
Vol vm.
?June 14th, 1890.
sZ?' voi- VJJLL?^une -^h, 1890.
at the General Post Office as a Newspaper for
^Bion in the United Kingdom and Abroad. J
H
Per Annum; 6s. 6d.; post free.
Monthly Parts, 6<L; post free 8d,
Ditto per Annum, 8s., post free.
Skirntt, /Ifteirictm, IFlurstitg aitb flbljtlatttfjtopg.
a\|

				

